# Lung recovery from DNA damage induced by graphene oxide is dependent on size, dose and inflammation profile

**DOI:** 10.1186/s12989-022-00502-w

**Published:** 2022-09-21

**Authors:** Luis Augusto Visani de Luna, Thomas Loret, Alexander Fordham, Atta Arshad, Matthew Drummond, Abbie Dodd, Neus Lozano, Kostas Kostarelos, Cyrill Bussy

**Affiliations:** 1 Nanomedicine Lab 2.0, Faculty of Biology, Medicine and Health, Manchester Academic Health Science Centre, The University of Manchester, Manchester, M13 9PT UK; 2 National Graphene Institute, The University of Manchester, Manchester, M13 9PL UK; 3 Lydia Becker Institute of Immunology and Inflammation, Faculty of Biology, Medicine and Health, Manchester Academic Health Science Centre, The University of Manchester, Manchester, M13 9PT UK; 4 Catalan Institute of Nanoscience and Nanotechnology (ICN2), CSIC and BIST, Campus UAB, Bellaterra, 08193 Barcelona, Spain

**Keywords:** Lungs, Graphene oxide, Genotoxicity, γ-H2AX, Inflammation, Toxicology

## Abstract

**Background:**

A key aspect of any new material safety assessment is the evaluation of their in vivo genotoxicity. Graphene oxide (GO) has been studied for many promising applications, but there are remaining concerns about its safety profile, especially after inhalation. Herein we tested whether GO lateral dimension, comparing micrometric (LGO) and nanometric (USGO) GO sheets, has a role in the formation of DNA double strand breaks in mouse lungs. We used spatial resolution and differential cell type analysis to measure DNA damages in both epithelial and immune cells, after either single or repeated exposure.

**Results:**

GO induced DNA damages were size and dose dependent, in both exposure scenario. After single exposure to a high dose, both USGO and LGO induced significant DNA damage in the lung parenchyma, but only during the acute phase response (*p* < 0.05 for USGO; *p* < 0.01 for LGO). This was followed by a fast lung recovery at day 7 and 28 for both GOs. When evaluating the chronic impact of GO after repeated exposure, only a high dose of LGO induced long-term DNA damages in lung alveolar epithelia (at 84 days, *p* < 0.05). Regardless of size, low dose GO did not induce any significant DNA damage after repeated exposure. A multiparametric correlation analysis of our repeated exposure data revealed that transient or persistent inflammation and oxidative stress were associated to either recovery or persistent DNA damages. For USGO, recovery from DNA damage was correlated to efficient recovery from acute inflammation (*i.e*., significant secretion of SAA3, *p* < 0.001; infiltration of neutrophils, *p* < 0.01). In contrast, the persistence of LGO in lungs was associated to a long-lasting presence of multinucleated macrophages (up to 84 days, *p* < 0.05), an underlying inflammation (IL-1α secretion up to 28 days, *p* < 0.05) and the presence of persistent DNA damages at 84 days.

**Conclusions:**

Overall these results highlight the importance of the exposure scenario used. We showed that LGO was more genotoxic after repeated exposure than single exposure due to persistent lung inflammation. These findings are important in the context of human health risk assessment and toward establishing recommendations for a safe use of graphene based materials in the workplace.

**Supplementary Information:**

The online version contains supplementary material available at 10.1186/s12989-022-00502-w.

## Introduction

Inhalation of airborne particulate matter has been linked to long-term pulmonary adverse effects and diseases, including asthma, susceptibility to infections, chronic obstructive disease, genotoxicity and cancer [1–4]. For the latter, it has been established that any particle or substance that has the ability to cause increased and long-lasting DNA damages with inefficient repair could induce genomic instability, mutation hotspots and finally tumor [5]. More recently, both air pollution and occupational health studies have stressed the potential of airborne carbon-based particles to give rise to genotoxicity and carcinogenicity [6]. And with the expansion of nanotechnologies and advanced materials, similar concerns have been raised for engineered carbon nanomaterials (CNMs) [7, 8].

Among those CNMs, carbon nanotubes (CNTs) are by far the most studied. While some CNTs were not found to cause any toxicity, others were reported to induce a large range of adverse effects, including cytotoxicity, inflammation, genotoxicity and cancer [9, 10]. In the lungs, some CNTs were shown to elicit frustrated phagocytosis in macrophages [11], to have poor biodegradability and high tissue biopersistence [11, 12], or to induce fibrosis [13, 14], mutagenesis [10] and carcinogenesis [15, 16]. When comparing these effects with those of asbestos fibers, it became apparent that shape and size along with other variables (*i.e*., diameter, rigidity, composition, surface chemistry, or metal impurities) were key drivers of CNT toxicity [12, 17].

In the last 15 years, another type of CNMs with large industrial potential has appeared, namely graphene based materials. In particular, graphene oxide (GO), the oxidized form of graphene, or its reduced form (rGO), have potential application in different sectors of the economy including the biomedical industry [18–21]. GO can be formulated either as powder or as aqueous suspension of two dimensional sheets, in a wide range of sizes (from micrometers to nanometers) and with varied degrees of surface oxidation. Interestingly, due to their size and shape, two dimensional GO sheets can easily form aerosols [22–25]. For this reason and considering the toxicological profile of others CNMs, in particular the genotoxicity and carcinogenicity of some CNTs, efforts have been made to evaluate the impact of GO or rGO to the lungs after inhalation [7, 26–30].

While there are several toxicology studies reporting the genotoxic potential of GO on lung cells using different models and in vitro settings, in vivo pulmonary investigations on this topic remain scarce [7]. Our group has previously shown that a high dose of micrometric GO delivered to the lungs of mice by single intranasal aspiration can lead to the activation of molecular pathways associated to genotoxicity or tumor development [27]. In a study directly investigating the genotoxicity of GO after single pulmonary exposure in mice, Bengtson et al. have reported that significant DNA damages could be found in cells from the broncho-alveolar fluid (BALF), but not in lung or liver tissue sections [28]. In a follow-up study, the same authors confirmed the outcomes in lung and liver at mRNA level [29].

Although these different studies demonstrated that GO may have a genotoxic impact on some lung cells after inhalation, they remain limited in their design by the use of single/bolus exposure. Chronic exposure and long-term assessment represent a more realistic scenario to evaluate persistent DNA damages that may lead to carcinogenesis events [16]. In this respect, we recently demonstrated that a repeated pulmonary exposure to GO via oro-pharyngeal aspiration induced a size, dose and time dependent lung inflammation without tissue remodeling in mice [26]. Importantly, we determined that these effects and their progressive disappearance were associated with a size-dependent lung clearance of the GO materials [26]. However, long-lasting inflammation such as the one observed with micrometric GO [26] has been previously associated with an increased risk of developing cancer [31]. There is therefore a need to evaluate the potential of these micrometric materials to cause genotoxicity after chronic exposure, and the possible link between inflammation and DNA damage.

In pulmonary hazard assessment studies, achieving an even distribution and deposition of nanomaterials in the lungs is known to be challenging. This is primarily due to the tendency of nanomaterials, including GO sheets, to agglomerate while progressing through the airways, from the upper bronchial ramifications down to the alveolar cavities [10, 32, 33]. As a consequence of agglomeration, nanomaterials accumulate in greater amount in various but discrete parts of the lungs (in both lung parenchyma and airways) where they trigger region-specific adverse effects, as previously described for silica particles, asbestos fibers or carbon nanotubes [14, 34]. Adverse effects initiated from these local areas of greater accumulation then spread across the whole tissue via cell signaling and/or the secretome. At tissue level, the overall/average effect of such nanomaterials is therefore dominated by how intense the effects were in regions of greater accumulation and balanced out by the limited to negligible response from the remaining tissue, where materials accumulated less. From this it follows that whole tissue analysis, which is often the default approach to probe the effects of nanomaterials, tends to provide a skewed picture and underestimates how adverse the effects of nanomaterials can be locally (*i.e*., dilution of the local effect in an average tissue level assessment). This is particularly true for genotoxicity that may lead to malignant transformation from events happening in a single/small group of cells and not in the whole tissue. Moreover, even for whole tissue analysis, there is still debate about the applicability of current OECD recommended methods (*i.e.*, TG 489, comet assay or TG 488, transgenic rodents) to determine DNA damages elicited by nanomaterials in vivo [35, 36]*.* Conversely, we argue that methods based on local analysis of specific lung regions could be advantageous compared to whole organ analysis for the assessment of nanomaterial genotoxicity, as they provide highly valuable spatial information. For instance, using immunostaining and γ-H2A.X (S139 phosphorylated H2AX) as marker for DNA double strand breaks (DSB) [37, 38] may provide better spatial insights to evaluate the genotoxicity of GO in lungs after pulmonary exposure. In particular, immunostaining used in a multiplex format (*i.e*., to reveal other protein biomarkers) or in combination with correlative imaging may help to determine the spatial distribution of DNA damages within the lungs while simultaneously identify the cells affected by these damages.

With this in mind, the primary aim of the present study was to reveal whether GO could induce long-lasting DNA damages in the lungs of mice, and whether these effects align with previous evidence of inflammation and oxidative stress [26]. To get a better mechanistic understanding of GO effects, we designed the study to evaluate not only the role of dose and time but also lateral dimension and exposure regimen. We compared the impact of single exposure to a high-dose (30 µg) of GO, to the impact of repeated exposure to two doses (cumulated low dose 3 µg or cumulated high dose 30 µg) of GO, using micrometric GO (LGO) and nanometric GO (USGO) in both cases. Impact of single oro-pharyngeal aspiration were assessed for up to 28 days after exposure, whereas the impact of repeated exposure was assessed for up to 84 days. All results were compared with the corresponding impact of long and rigid MWCNTs (Mitsui-7) at the same time points and doses. To get spatial information about DNA damages and avoid the dilution effect inherent to whole tissue analysis, we used immunostaining of lung sections and quantified the level of genetic material alterations under the different conditions. Moreover, we used co-immunostaining to reveal the identity of cells presenting DNA damages in the lung sections (*i.e*., epithelial or immune cells), and correlative imaging to identify the location of DNA damages in respect to the cell mediated immune response within the lungs. To complement these image-based techniques, we finally used a multi-endpoint approach to correlate new data on DNA repair generated by RT-qPCR with our published data on lung inflammation and oxidative stress induced by repeated exposure to GO [26].

## Results and discussion

**Fig. 1:**
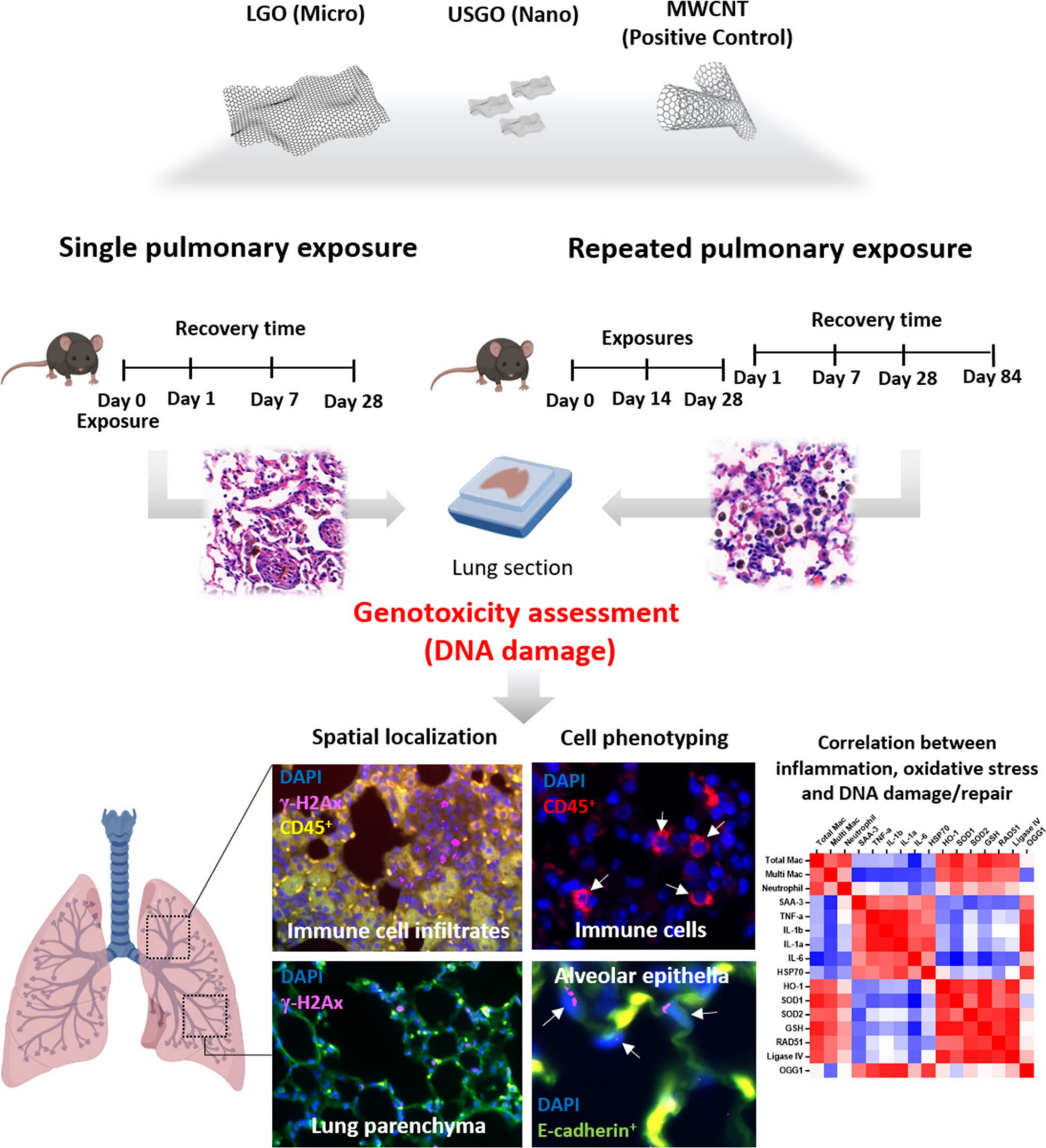
Experimental design of the study. Single (high-dose) and repeated (low and high-dose) exposure to nanometric (USGO) or micrometric (LGO) graphene oxide sheets or MWCNTs were delivered to mouse lungs by oro-pharyngeal aspiration. Quantification of DNA damages in formalin-fixed paraffin embedded lung sections was performed using recombinant rabbit anti-mouse γ-H2Ax-Phosphorilated Ser139, combined with anti-mouse CD45 Alexa Fluor 594 and anti-mouse E-Cadherin Alexa Fluor 488 to phenotype the damaged cells (*n* = 3). A correlation matrix was performed for the repeated exposure (high-dose) study using inflammation parameters obtained from BALF, whole lung ELISA, and RT-qPCR obtained from the same animals (*n* = 6). Figure created with BioRender.com

In the first part of the present study (Fig. 1), we evaluated the potential genotoxicity of GO sheets after single (30 µg) or repeated (3 × 1 µg or 3 × 10 µg) pulmonary exposure. The low dose of 3 × 1 µg represented a realistic dose of exposure at the workplace when limited mitigating measures are applied, whereas 30 µg (or 3 × 10 µg) was used here as worst-case exposure scenario (accident) that may happen during specific tasks such as dry powder handling without protective measures [39, 40]. The aim was to compare outcomes from standard exposure protocol (i.e., single exposure triggering acute response) to outcomes from chronic exposure that may better represent exposure at the workplace [26]. This would help to estimate whether the more practical and faster single exposure protocol can predict with enough accuracy the genotoxicity of GO or is missing out on subtle effects only appearing with chronicity. To address the role of time, we investigated the DNA damages at 1, 7 and 28 days after single exposure and 1, 7, 28 and 84 days after repeated exposure.

Building on our previous works [26, 27, 41], we also interrogated whether lateral dimensions of the GO sheets could be a key factor of their lung genotoxicity. Hence we compared micrometric LGO sheets (1–25 µm) with nanometric USGO sheets (10–300 nm) that had both similar thickness (1–2 nm) and the same physicochemical properties (Additional file 1: Fig. S1, Table S1) [42]. Both LGO and USGO suspensions were made of thin, endotoxin-free and metal-free GO sheets that have been fully characterized previously [26]. Importantly, a previous study has determined that 0.1 µm thick graphene based materials of 25 µm lateral size would have an aerodynamic diameter of 2.97 µm, well below the 5 µm threshold for reaching the alveolar region [43], suggesting that both LGO and USGO used here may reach the distal lung. In fact, we demonstrated in our previous work that about 85% of alveolar macrophages present in the broncho-alveolar lavage had engulfed GO materials 1 day after pharyngeal aspiration and that it represented about 60% of the macrophages’ internalization capacity at day 1 for both USGO and LGO exposures [26]. This result underlines that despite clear differences in size, similar amounts of either USGO or LGO could reach the alveolar space [26]. This similarity allowed in return an accurate evaluation of the impact of GO lateral dimension on lung genotoxicity. In parallel, multi-walled CNTs (MWCNTs, Mitsui-7) were used in both exposure scenarios as a positive control for the intended biological endpoints, based on the extensive number of reports in the literature demonstrating their genotoxicity [16, 44, 45].

The second part of the study aimed to reveal whether DNA repair mechanisms were triggered alongside the DNA damages. To explain the mechanisms behind the DNA damages or their recovery, we correlated the present results with our already published data on inflammation and oxidative stress caused by repeated exposure to high doses of GO sheets [26] (Fig. 1).

### Lung DNA damages induced by pulmonary exposure to GO depend on size, dose, time, and exposure regimen

First, potential DNA damages caused by GO after either single or repeated pulmonary exposure were investigated in lung sections using γ-H2A.X (S139 phosphorylated H2AX) immunostaining. We considered in our analysis either the total intensity of γ-H2A.X per field of view (FOV), which represents the overall/global DNA damage irrespective of cell type (i.e., it includes immune cell infiltrates, granulomatous structures and remaining lung parenchyma), or the number of γ-H2A.X positive nuclei in the non-inflammatory areas of the lung parenchyma (i.e., it excludes inflammatory areas). To get spatial insights and a better understanding of the cell types that may be affected by material-induced DNA damages, we performed co-immunostaining for γ-H2A.X (DNA damage marker) and CD45 (immune cell marker) or E-Cad (epithelial cell marker). This differential analysis was particularly important because genetic damages in epithelial cells can induce loss of proliferative control, and potentially lead to neoplastic lesions [3].

#### Location and cell type dependent DNA damages after single exposure

**Fig. 2:**
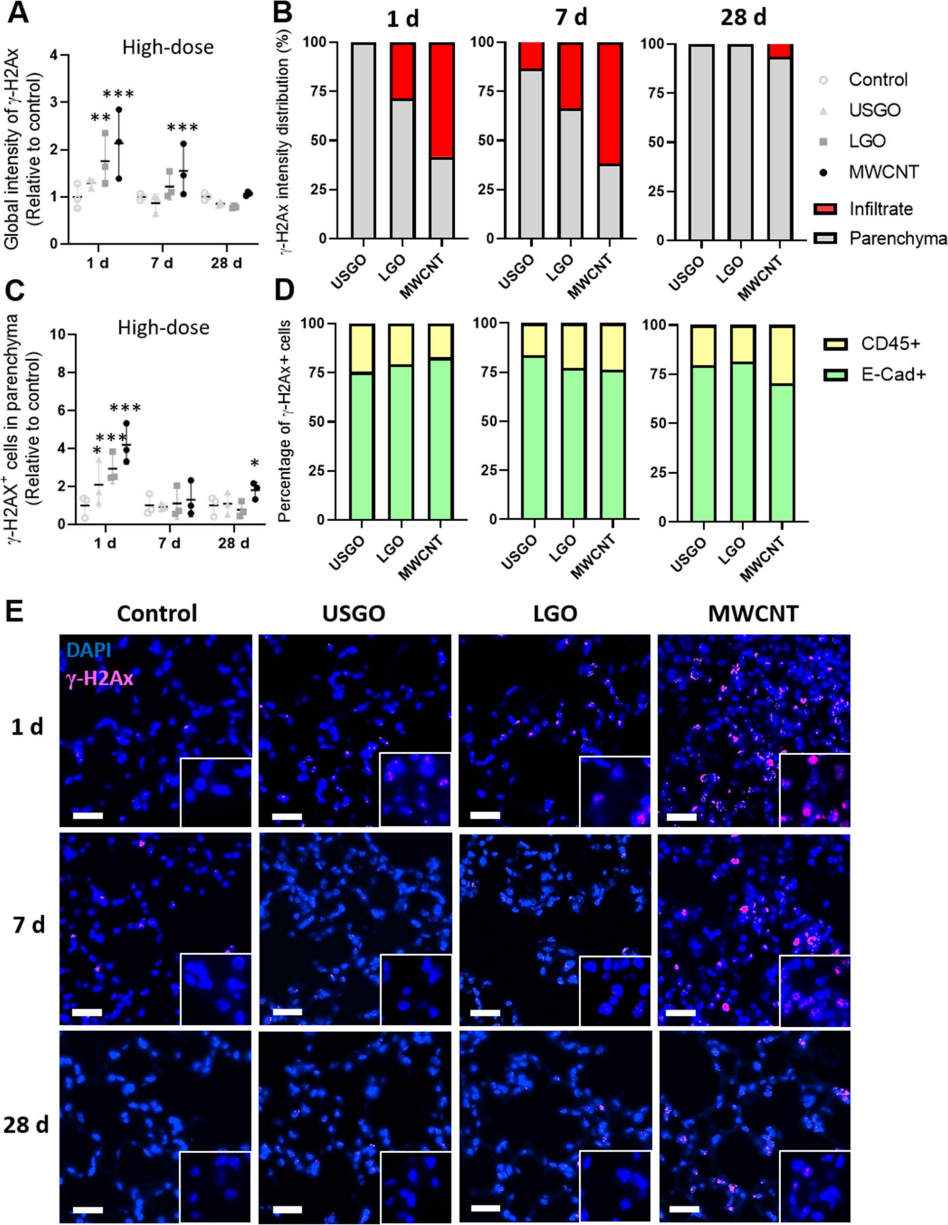
Evaluation of DNA damages induced by GO sheets after single exposure. **A** Quantification of lung DNA damages using rabbit anti-mouse γ-H2Ax-Phosphorilated Ser139 immunostaining after single exposure to 30 µg of GO sheets (USGO or LGO) and MWCNTs (positive control/reference materials) expressed as fold change in fluorescence intensity (negative control = water for injection). **B** Percentage of γ-H2Ax imunoreactivity (fluorescence intensity) located in lung immune cell infiltrates or in lung parenchyma at day 1, 7 and 28 after exposure. **C** Total number of γ-H2Ax positive cells in lung parenchyma (outside inflammatory infiltrates) expressed as fold change in fluorescence intensity. **D** Percentage of E-cadherin^+^ γ-H2Ax^+^ cells and CD45^+^ γ-H2Ax^+^ cells in lung parenchyma. **E** Representative images of lung section after DAPI staining and immunostaining (primary: rabbit anti-mouse γ-H2Ax-Phosphorilated Ser139; secondary: donkey anti-rabbit Alexa Fluor 647) for DNA damages. Mice were exposed by single oro-pharyngeal aspiration to a high dose GO sheets (USGO or LGO), MWCNTs, or water for injection. Scale bar = 50 µm. Significance level *p < 0.05 **p < 0.01, ***p < 0.001 (One-Way ANOVA; *n* = 3)

When performing global analysis of γ-H2A.X immuno-reactivity (i.e., total fluorescence intensity for γ-H2A.X; Fig. 2A, E), a single exposure to GO was found to cause significant DNA damages in lungs, but only at day 1 and for LGO. In contrast, no significant increase in global DNA damages could be measured in USGO exposed animals at any time point, highlighting that DNA damages were GO size dependent. When looking at the spatial location of the DNA damages, it is worth noting that a substantial amount of damages induced by LGO were concentrated in areas with immune cell infiltrates (Figs. 1 and 2B), visible in H&E stained lung sections (Additional file 1: Fig. S2), and represented by a high density of CD45^+^ cells (Fig. 1). Up to 28.5% of the global fluorescence intensity for LGO induced γ-H2A.X positive signal could be ascribed to these immune cell clusters at day 1 (i.e., 71.5% of the remaining LGO induce DNA damages were in non-inflammatory areas), increasing to 33.5% at day 7, but disappearing by day 28 (Fig. 2B). Although we did not observe any significant increase in DNA damage for USGO, it is worth mentioning that most of the DNA strand breaks events recorded were in the parenchyma (Fig. 2B). On the other hand, DNA damages induced by MWCNTs that were significant at both days 1 and 7 (Fig. 2A, E) were concentrated primarily in inflammatory areas at early time points (58.3% at day 1 and 61.7% at day 7; Fig. 2B) and in non-inflammatory areas at the latest time points (93.6% at day 28; Fig. 2B).

Focusing on γ-H2A.X immuno-reactivity in non-inflammatory areas of the lung parenchyma, we found a statistically significant increase in the number of γ-H2A.X positive cells only at 1 day and for both USGO and LGO (Fig. 2C). Importantly, a majority of these DNA damages were found in lung epithelial cells, corresponding to 75.3% and 79.2% for USGO and LGO respectively (Fig. 2D), as demonstrated using co-immunostaining (E-cad^+^; Additional file 1: Fig. S3A and CD45^+^; S3B). However, at day 7 and 28, neither LGO nor USGO were causing significant DNA damages in non-inflammatory areas, suggesting that DNA repair may have happened between day 1 and day 7, or there was clearance of DNA damaged cells. For MWCNTs, there was a statistically significant increase in the number of γ-H2A.X positive cells in non-inflammatory areas at both day 1 and day 28 in comparison to the negative control (Fig. 2C). At day 1, 82.8% of these DNA damages were found in lung epithelial cells; decreasing to 76.2% at day 7 and 70.5% at day 28 (Figs. 2D and Additional file 1: S3A), suggesting persistent damages to the epithelium or at least longer lasting damages compared to GO. Taken together, these data suggested that an efficient lung recovery, leading to a rapid eradication of DNA damages, coupled with a fast resolution of the acute inflammation (evidenced by H&E staining, Additional file 1: Fig. S2), took place early on in the lungs after exposure to GO. However, this was not the case after exposure to MWCNTs.

#### Location and cell type dependent DNA damages after repeated exposure

**Fig. 3:**
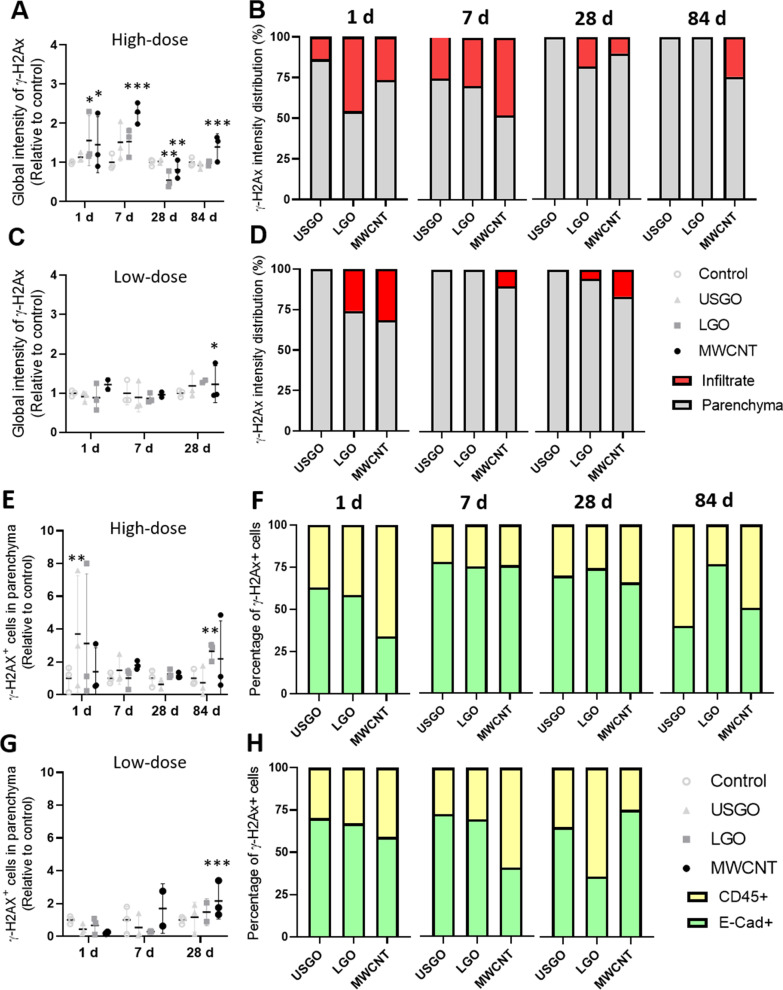
Evaluation of DNA damages induced by GO sheets after repeated exposure. **A** Quantification of lung DNA damages using rabbit anti-mouse γ-H2Ax-Phosphorilated Ser139 immunostaining after repeated exposure to 3 × 10 µg (high dose) of GO sheets (USGO or LGO) and MWCNTs (positive control/reference materials), expressed as fold change in fluorescence intensity (negative control = water for injection). **B** Percentage of γ-H2Ax imunoreactivity (fluorescence intensity) located in lung immune cell infiltrates or in lung parenchyma at day 1, 7, 28 and 84 after repeated exposure to 3 × 10 µg. **C** Quantification of lung DNA damages after repeated exposure to 3 × 1 µg (low dose) of materials. **D** Percentage of γ-H2Ax imunoreactivity (fluorescence intensity) located in lung immune cell infiltrates or in lung parenchyma at day 1, 7 and 28 after repeated exposure to 3 × 1 µg. **E** Total number of γ-H2Ax positive cells in lung parenchyma (outside inflammatory infiltrates) expressed as fold change in fluorescence intensity, after repeated exposure to 3 × 10 µg. **F** Percentage of E-cadherin^+^ γ-H2Ax^+^ cells and CD45^+^ γ-H2Ax^+^ cells in lung parenchyma at day 1, 7, 28 and 84 after exposure. **G** Total number of γ-H2Ax positive cells in lung parenchyma (outside inflammatory infiltrates) expressed as fold change in fluorescence intensity, after repeated exposure to 3 × 1 µg. **H** Percentage of E-cadherin^+^ γ-H2Ax^+^ cells and CD45^+^ γ-H2Ax^+^ cells in lung parenchyma at day 1, 7 and 28 after exposure. Significance level *p < 0.05 **p < 0.01, ***p < 0.001 (One-Way ANOVA; *n* = 3)

**Fig. 4:**
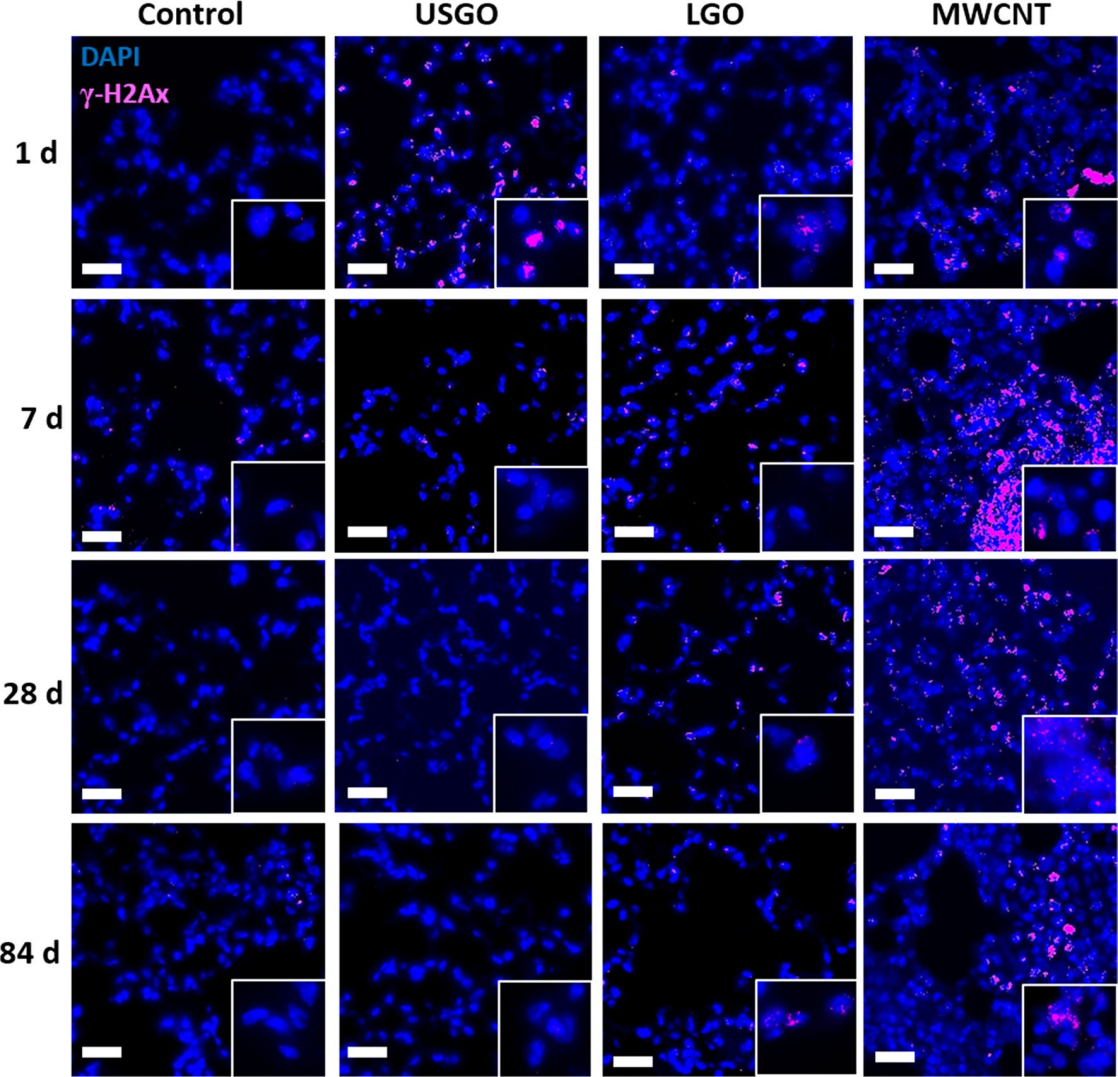
Representative images of immunostaining for DNA damages in lung sections after repeated exposure to a high dose of materials. DNA damages was performed using rabbit anti-mouse γ-H2Ax-Phosphorilated Ser139, and donkey anti-rabbit Alexa Fluor 647. Mice were exposed by single oro-pharyngeal aspiration to a high dose GO sheets (USGO or LGO), MWCNTs, or water for injection. All sections were counterstained for DAPI. Scale bar = 50 µm. Inset boxes highlight positive nuclei with higher magnification (100×)

After repeated exposure to GO, we found that only the high dose of LGO induced a statistically significant increase in DNA double strand breaks in the lungs, and only 1 day after the last exposure (Figs. 3A, B and 4 for high dose; 3C and 3D for low dose). This suggests that GO genotoxicity was both dose and time dependent. Importantly, irrespective of the time point, neither LGO nor USGO induced significant DNA damages at the low dose (1 µg repeated 3 times) (Fig. 3C, D), which represents a realistic dose relevant to human exposure at the workplace [39, 40]. When looking at the location of LGO induced damages at the high dose, 45.8% of them were located in inflammatory areas (Fig. 3B). These results suggest that GO-induced immune cell infiltrates, which size increased with the dose applied or the dimension of the material used [26], may be areas with higher DNA damage prevalence. This agrees with previous studies reporting DNA damages in immune cells collected from BALF but not in lungs after pulmonary exposure to GO [28]. This may be due to the ability of these immune cells to rapidly phagocyte nanomaterials that may have genotoxic effects, hence preventing damage to lung epithelial cells. This is also in line with previous work showing genotoxicity in lung overload condition, when clearance by phagocytes is impaired due to an excess of nanomaterial in the lungs, but no genotoxicity in a non-overload condition [46].

Interestingly, the DNA damages found in the immune cell infiltrates were alleviated over time, dropping to 29.8% at day 7, then 18.3% at day 28, and disappearing entirely by day 84 (Fig. 3B). This suggests some recovery (i.e., DNA repair) and/or clearance of the damaged (immune) cells from the lungs. Immune cells called to resolve an inflammatory event in lungs are known to reverse migrate to other locations after inflammation has vanished [47]. The decrease in LGO induced DNA damages over time (Fig. 3B), which is aligned with the resolution of the inflammation over time [26], might hence be explained by the relocation of damaged cells to extra-pulmonary locations. Future research should aim at investigating this possibility in expected locations, such as lymph nodes or bone marrow.

Finally and as expected, high dose of MWCNTs induced significant DNA double strand breaks at any of the tested time points (Figs. 3A and 4), affecting primarily immune cell infiltrates (Fig. 3B). These results are consistent with the persistence of immune cell infiltrates, for up to 84 days after the last exposure to MWCNTs [26], where the materials are accumulated. In contrast, low dose MWCNTs induced statistically significant DNA damages only at day 28 and primarily in the lung parenchyma (Fig. 3C, D), which could be explained by the lower accumulation of materials in immune infiltrates at the low dose.

Focusing on γ-H2A.X immuno-reactivity in non-inflammatory areas of the lung parenchyma, we found using co-immunostaining that a high dose of USGO induced significant DNA damages at day 1 (Fig. 3E) and mostly in alveolar epithelial cells (E-Cad^+^; 63.1%; Figs. 3F and Additional file 1: S4A). There was no other statistically significant result for USGO at any other time point or in the low dose group (Fig. 3G, H and Additional file 1: Fig. S4), suggesting that USGO induced DNA damages in these cells were both time and dose dependent. For LGO, we found significant DNA damages only in the high dose group and at 84 days after the last exposure (Fig. 3E), with 76.4% of the damages found in alveolar epithelial cells (Figs. 3F and Additional file 1: Fig. S4A). Nevertheless, when evaluating DNA damages only in the epithelial cells (E-Cad +), there was also some significant DNA damages at day 1 (Additional file 1: Fig. S4). Importantly, there was no significant DNA damages at the low dose (Fig. 3G, H and Additional file 1: Fig. S4B), suggesting that LGO induced DNA damages in epithelial cells are mostly dose dependent. The fact that a high dose of LGO can induce DNA damages in lung epithelial cells, not only at an early time point after exposure but also at a late stage of the washout period (here 3 months after last exposure; Additional file 1: Fig. S4A), raises some concerns about the long(er) term impact of these DNA damages, and warrant further research to address these questions.

On the other hand, and surprisingly for MWCNTs, there was no statistically significant DNA damages at the high dose in non-inflammatory areas (Fig. 3E, F); significance was found only at 7 days when isolating the results obtained for lung epithelial cells (E-Cad + ; Additional file 1: Fig. S4A). However, in agreement with the results reported above (using global analysis that includes immune cell infiltrates, granulomatous structures and lung parenchyma; Fig. 3C), we found significant DNA damages at day 28 for the low dose (Fig. 3G) and most of them were located in alveolar epithelial cells (75%; Figs. 3H and Additional file 1: Fig. S4B). This could be explained by a lower agglomeration of these materials at low dose, allowing them to diffuse more easily throughout the parenchyma, and not being entrapped in immune cell infiltrates.

#### Potential genotoxicity of carbon nanomaterials in lungs

Overall, the above results suggest that lungs were able to recover rapidly (within 7 days) from exposure to GO sheets with nanometric dimensions even after multiple exposures, whereas exposure to GO sheets with micrometric dimensions may cause long-term genotoxic effect. Noticeably, the latter result was only perceivable in the repeated exposure at the latest time point after exposure, namely 84 days, highlighting the value of chronic exposure over acute exposure to reveal subtle long–term changes. Beyond size, these results further demonstrate that dose (‘high’ worse than ‘low’) and exposure regimen (‘repeated’ worse than ‘single’) are critical factors to consider when assessing the potential genotoxic impact of GO in lungs, especially for alveolar epithelial cells (Additional file 1: Figs. S3A, S4). In contrast to these findings, Bengtson et al*.* have previously shown using comet assay on cell isolates from whole tissue that a single dose of GO (18 µg) delivered to mice by intratracheal instillation could not induce significant DNA damages in lung or liver [28]. Exploring the transcriptomic differences in whole lung and liver tissues after pulmonary exposure to either GO or reduced GO (from 18 to 162 µg/mouse), they confirmed that there was no significant activation of genotoxicity pathways in lung tissue [29]. Interestingly and in agreement with some of our present results, they also demonstrated that despite the lack of DNA damages in whole lungs, (immune) cells collected from lung BAL displayed GO-induced genotoxic effects [28]. However, further comparison with the present study are limited because of the differences in methods used to evaluate genotoxicity or the physicochemical features of the tested GO materials. In the same study, Bengtson et al*.* reviewed the lung genotoxicity profile of other carbon nanomaterials and reported that reduced GO was also not inducing significant DNA damages to lungs [28].

Conversely, carbon black (18–162 µg) was shown to induce significant DNA double strand breaks in BAL cells and lungs for up to 28 days after single intratracheal instillation [48]. The authors emphasized the contribution of a persistent inflammation in lungs, especially with the increased expression of SAA3 at all time-points (i.e., 1, 3 and 28 days after exposure). They further correlated the measured DNA double strand breaks with oxidative DNA damages and inflammation levels, and concluded that the lung genotoxicity of carbon black was both oxidative stress and inflammation dependent [48]. Similarly, Kato et al*.* demonstrated in mice that MWCNTs (50–200 µg, Mitsui-7, same MWCNTs as in the present study) induced significant DNA damages in lungs at 3 h after instillation, and significant oxidative DNA damages in lungs at any tested time point (up to 7 days) [49]. The authors also highlighted that these MWCNTs elicited mutagenicity in lungs of transgenic mice *(gpt* delta, used for rodent gene mutation assays, TG488 [35]), but only after multiple instillation to the highest dosage (4 × 200 µg). Going further, Kasai et al*.* demonstrated that chronic exposure to MWCNT (Mitsui-7) aerosols led to lung carcinogenicity in rats [15].

In the present study, a majority of the DNA double strand breaks induced by GO after either single or repeated exposure was in alveolar epithelial cells (Figs. 2 and 3). While there is a relative paucity of in vivo studies on the genotoxicity of GO, the number of predictive in vitro studies based on lung epithelial cell models is more substantial. Moreover, despite the limitations and differences in terms of dose applied or model used when comparing in vivo and in vitro data [50, 51], in vitro models may provide more detailed insights to the mechanisms leading to genotoxicity. In murine pulmonary epithelial cell line FE1, GO (5–200 µg/mL) did not induce significant DNA damages after 24 h of exposure, although strong ROS generation was reported [52]. In both alveolar epithelial A549 and bronchial epithelial BEAS-2B models, GO (10–100 µg/mL) induced significant micronuclei formation after 6 h of exposure in a dose-dependent manner, and significant ROS production at the highest dose tested [53]. Alongside direct DNA damages due to the interaction of nanomaterials with the genetic materials, it is well-known that DNA damages may result from bystander effects due to nanomaterials (i.e., inflammation mediated ROS level). This type of DNA damages, called secondary genotoxicity [54], has been recently reported for graphene nanomaterials in human-transformed type-I (TT1) alveolar epithelial cell [55]. In this in vitro study, both inflammation and oxidative stress were the two factors associated with either transient or persistent DNA damages. In another study using the BEAS-2B model, both single- and few- layer GO sheets (50 µg/mL) induced significant DNA damages after 24 h of exposure, reduction in LIG4 (DNA ligase 4) expression, but no variation in RAD51 (DNA repair protein RAD51 homolog 1) expression; while a significant increase in OGG1 (8-oxoguanine DNA glycosylase) expression was noticed for single-layer GO only [56].

In summary, these different in vitro data confirm that GO and other graphene based materials can cause DNA damages in lung epithelial cells and that these effects are often associated with oxidative stress and inflammation. In fact, both in vivo and in vitro data existing for either GO or other carbon nanomaterials suggest that genotoxicity is often primarily due to the unbalance between a high ROS level typically found in an inflammatory environment, and the anti-inflammatory, anti-oxidant and DNA repair defense mechanisms trying to counteract the effects of inflammation and ROS. This led us to seek whether the spatially-resolved DNA damages found here with GO could be correlated to the induction of DNA repair mechanisms and to previously published in vivo data on inflammation and oxidative stress after repeated exposure to GO.

### Lung DNA damages induced by repeated pulmonary exposure to GO are correlated to inflammation, oxidative stress, and DNA repair events

**Fig. 5:**
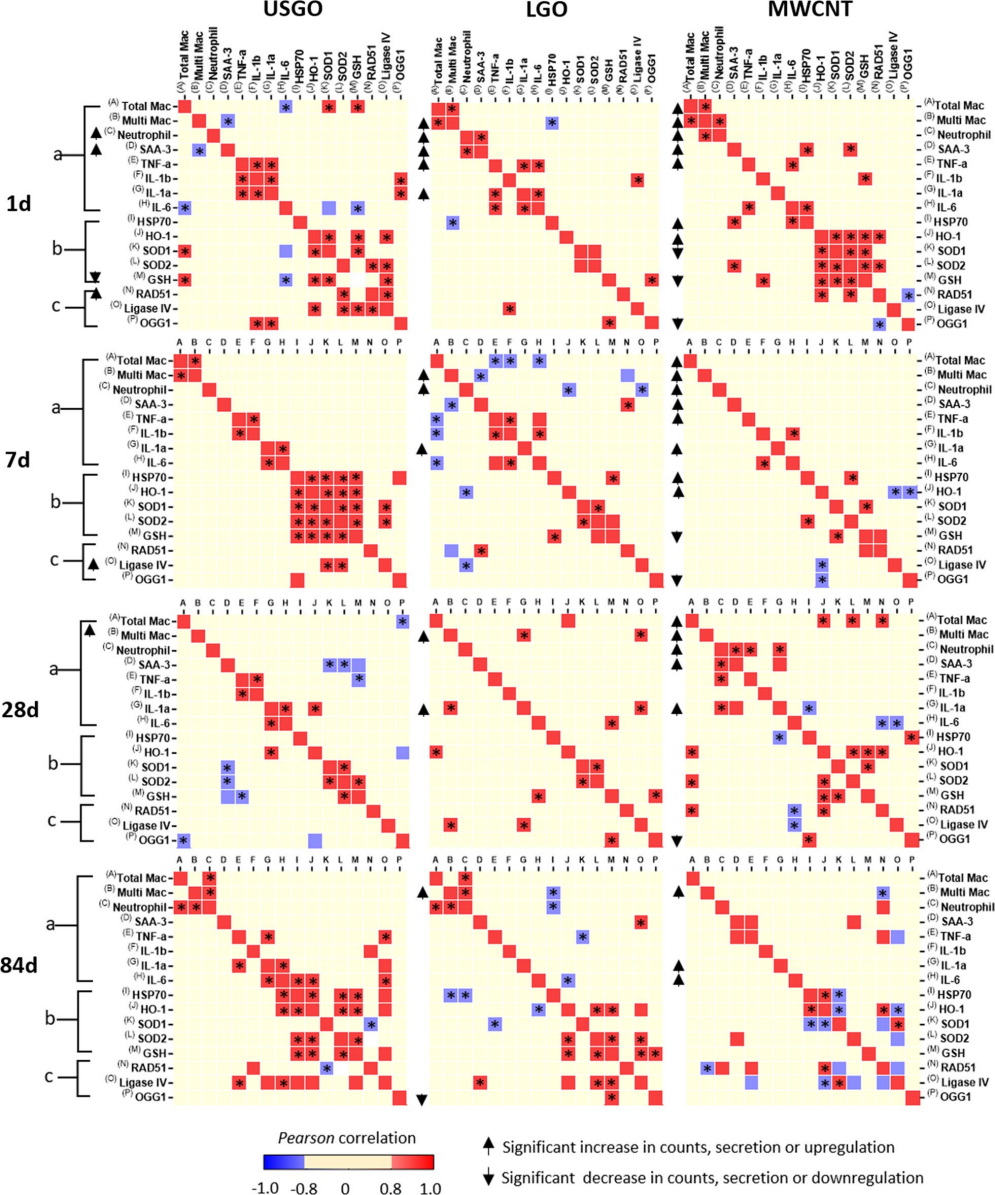
Correlation matrix of inflammation, oxidative stress and DNA repair in lungs of mice repeatedly exposed by oro-pharyngeal aspiration to a high dose of GOs or MWCNTs. Data of multiple biomarkers were gathered and then transformed in log2 of fold change compared to negative control (water for injection) before the *Pearson* correlation analysis was performed. Group (a) represents the inflammation markers: with cellular markers determined from BALF analysis [26] (A = Total Mac (number of macrophages); B = Multi Mac (number of multinucleated macrophages); C = Neutrophils (number of Neutrophils)) and with pro-inflammatory cytokines detected by ELISA in lung tissue (D = Saa3; E = TNF-α; F = IL-1β; G = IL-1α; H = IL-6). Group (b) represents oxidative stress markers (I = HSP70, J = HO-1, K = SOD1, L = SOD2 and M = GSH) assessed by RT-qPCR [26]. Group (c) represents DNA repair proteins (N = RAD51, O = LIG4 and P = OGG1) assessed by RT-qPCR in whole lung tissue. Increased cell counts, secretion and upregulated expression are highlighted by upward arrows adjacent to the matrix, and decreased or downregulated are highlighted by downward arrows. *Pearson* positive correlation is highlighted by red squared boxes and negative correlation by blue squared boxes. Significance level of the correlation *p < 0.05. The *Pearson* values and their respective *p* values of significance are reported in tables in the supplementary content

To evaluate the extent of ongoing DNA repair in the lungs of mice repeatedly exposed to a high dose of either USGO or LGO, we assessed the mRNA expression level of three essential DNA repair proteins, namely RAD51, LIG4, and OGG1 (Additional file 1: Fig. S5). This first data set was then cross-correlated in a correlation matrix with a second data set based on our previously published data on lung inflammation and oxidative stress induced by GO [26] (Additional file 1: Figs. S6, S7) in order to identify the possible cause of GO induced DNA damages (Fig. 5). In both cases, only the data related to repeated exposure to a high dose of USGO or LGO, and MWCNTs used as positive control, were considered. A similar strategy has been previously applied for MWCNTs in order to reveal which nanomaterial physicochemical features could predict pulmonary inflammation and genotoxicity [9, 57]. To confirm the spatial distribution of DNA damages with respect to inflammatory areas and the rest of the lung parenchyma, we then performed correlative imaging. For that, we correlated H&E staining (to reveal immune structures) and γ-H2A.X related DNA double strand breaks immunostaining at day 1 and 84 after repeated exposure to the different materials (Additional file 1: Fig. S8).

#### Correlation analysis after repeated exposure to a high dose of USGO

*Correlation with inflammation* At day 1 after exposure to USGO, we previously demonstrated that there was a significant secretion of pro-inflammatory cytokines and evidence of acute inflammation (i.e., significant increase in SAA3 expression level, Additional file 1: Fig. S6**;** [26]). However, despite a significant increase in expression of RAD51 mRNA level (Additional file 1: Fig. S5), this was not correlated to any inflammation event (Fig. 5). Nevertheless, there was a positive correlation (Fig. 5) between the mRNA expression level of OGG1 (decrease in expression compared to the control, although non-significant; Additional file 1: Fig. S5) and the mRNA expression levels of both IL1β and IL1α (non-significant increase for both; Additional file 1: Fig. S6). At day 7, there was neither a significant increase in immune cell infiltrates, nor significant secretion in pro-inflammatory cytokines. Despite a significant increase in expression of LIG4 mRNA level (Additional file 1: Fig. S5), this was not correlated to any inflammation event (Fig. 5). Increased expression levels of RAD51 at day 1 and LIG4 at day 7, which are both proteins able to repair DNA double strand breaks [58], suggest that DNA defense mechanisms were counteracting the negative effects on DNA found for high dose USGO in the non-inflammatory lung parenchyma areas (Fig. 3). At days 28 and 84, there was neither significant increase in inflammatory markers (including both recruited immune cells and secreted cytokines; group (a) in Fig. 5), nor significant increase in the expression of DNA repair proteins (Additional file 1: Fig. S5 and group (c) in Fig. 5), suggesting a successful resolution from both the initial mild inflammation and DNA damages observed in the lungs at day 1 after USGO treatment. However, we identified at day 84 a positive correlation (Fig. 5) between the mRNA expression level of LIG4 (non-significant decrease; Additional file 1: Fig. S5) and the mRNA expression levels of both TNFα and IL6 (non-significant increase for both; Additional file 1: Fig. S5), suggesting that lungs were still in a recovery phase.

*Correlation with oxidative stress* As mentioned above, there was a significant increase in the mRNA expression level of RAD51 at day 1 (Additional file 1: Fig. S5) that correlated positively with the expression level of LIG4 (non-significant decrease; Additional file 1: Fig. S5 and Fig. 5). More interestingly, both of these proteins were found to have a positive correlation with the expression level of SOD2 (non-significant increase; Additional file 1: Fig. S7 and Fig. 5). Noticeably for this time point, there was also a positive correlation (Fig. 5) between the mRNA expression level of LIG4 (not significant decrease; Additional file 1: Fig. S5) and the expression levels of GSH (significant decrease) and HO1 (non-significant decrease) (Additional file 1: Fig. S5). Although significant lung DNA damages were no longer detected at day 7 (Fig. 3), the significant increase in LIG4 mRNA expression level (Additional file 1: Fig. S5) was positively correlated with SOD1 and SOD2 expression levels (non-significant increase in both cases; Additional file 1: Fig. S7), highlighting the contribution of antioxidant enzymes in the DNA repair (Fig. 5). At day 28 and 84, there was no positive correlation between the expression levels of DNA repair proteins and oxidative stress markers (Fig. 5).

#### Correlation analysis after repeated exposure to a high dose of LGO

*Correlation with inflammation* At day 1, LGO induced a significant inflammation and the formation of multinucleated macrophages [26] (Additional file 1: Figs. S6, S7). However here, only the non-significant increase in mRNA expression of IL1β (Additional file 1: Fig. S5) correlated positively with the non-significant decrease in mRNA expression of LIG4 (Additional file 1: Fig. S5 and Fig. 5). At day 7, the LGO-induced inflammation, which included the presence of multinucleated macrophages in the lungs, was still above the level found for the negative control, despite evidence of resolution (Additional file 1: Fig. S6; [26]). Interestingly, there was a negative correlation (Fig. 5) between the mRNA expression level of RAD51 (undisturbed in comparison to the control; Additional file 1: Fig. S5) and the presence of multinucleated macrophages in BALF (significant increase; Additional file 1: Fig. S6). Although negative correlation was observed in this case, it is known that BALF cells might be sensitive to DNA double strand breaks [28, 54]. This was demonstrated in our above mentioned findings in which immune cell infiltrates were identified as hotspots of GO-induced DNA damages (Fig. 3B). This negative correlation can be explained by either poor ongoing DNA repair or limitations in the whole lung analysis (used here for determining the mRNA expression level), which is not efficient and sensitive enough to detect discrete DNA repairs happening locally at the site of DNA damages. On the other hand, a positive correlation was observed (Fig. 5) between the mRNA expression level of RAD51 (undisturbed in comparison to the control; Additional file 1: Fig. S5) and the expression level of SAA3 (undisturbed in comparison to the control; Additional file 1: Fig. S6). More importantly, there was a strong negative correlation (Fig. 5) between the mRNA expression of LIG4 (non-significant increase; Additional file 1: Fig. S5) and the presence of neutrophils in BALF (undisturbed in comparison to the control; Additional file 1: Fig. S6). This suggested no association between the ongoing inflammation due to LGO and this DNA repair enzyme (Fig. 5). At day 28, the presence of multinucleated macrophages and inflammatory mediators, such as IL1 α, in the lungs (statistically significant for both) was suggesting that the inflammation was still ongoing (Additional file 1: Fig. S6; [26]). Interestingly, we found a positive correlation (Fig. 5) between the mRNA expression level of LIG4 (non-significant increase; Additional file 1: Fig. S5) and the presence of macrophages as well as the increase in mRNA expression level of IL1α (Additional file 1: Fig. S6). At day 84, we only found a positive correlation (Fig. 5) between the mRNA expression level of LIG4 (non-significant increase; Additional file 1: Fig. S5) and the mRNA expression level of SAA3 (undisturbed in comparison to the control; Additional file 1: Fig. S6).

*Correlation with oxidative stress* OGG1 is a DNA repair enzyme associated to both inflammation and oxidative stress [59]. Its main role is the excision of oxidized guanine nucleotides, which can turn into DNA mutation hotspots if they are not eliminated. At day 1, we found a positive correlation (Fig. 5) between the mRNA expression level of OGG1 (not significant decrease; Additional file 1: Fig. S5) and the mRNA expression level GSH (non-significant decrease; Additional file 1: Fig. S7). This positive correlation between OGG1 and GSH was also present for day 28 and 84 (Fig. 5, Additional file 1: Figs. S5, S7). Interestingly at day 84, we also found a positive correlation (Fig. 5) between the mRNA expression level of LIG4 (non-significant decrease; Additional file 1: Fig. S5) and the expression levels of SOD2 and GSH (non-significant decrease; Additional file 1: Fig. S7). These decreases in DNA repair proteins and correlation with antioxidant enzymes at day 84 may explain the significant DNA damages found in mouse lungs at 84 days after exposure to high dose LGO (Fig. 3).

#### Correlation analysis after repeated exposure to a high dose of MWCNTs

*Correlation with inflammation* At day 1, there was a significant influx of immune cells in the BALF, with presence of multi-nucleated macrophages and pro-inflammatory cytokines, all revealing a strong inflammation (Additional file 1: Figs. S6, S7 and S8). From day 1 to 84, lung inflammation (Additional file 1: Figs. S6, S7; [26]) and DNA damages (Fig. 3A and Additional file 1: Fig. S8) after repeated exposure to MWCNTs were still significantly above the negative control levels. However, none of the DNA repair proteins were upregulated at any time points; in fact the mRNA expression level of OGG1 was decreased in comparison to the negative control (statistically significant decrease at day 1, 7 and 28, but not at day 84; Additional file 1: Fig. S5). Despite DNA damages (Fig. 3 and Additional file 1: Fig. S8) and strong immune response involving both innate and adaptive immune cells (Fig. 5; [26]), no correlation were found at day 1 and day 7 (Fig. 5). At day 28, there was a positive correlation (Fig. 5) between the mRNA expression level of RAD51 (not significant decrease; Additional file 1: Fig. S5) and the influx of macrophages (significant increase compared to control; Additional file 1: Fig. S6). Interestingly both RAD51 and LIG4 mRNA expression levels (Additional file 1: Fig. S5) were negatively correlated with the mRNA expression level of IL6 (non-significant increase; Additional file 1: Fig. S6). At day 84, RAD 51 expression level (Additional file 1: Fig. S5) was also negatively correlated (Fig. 5) with the presence of multinucleated macrophages in the BALF (Additional file 1: Fig. S6). The same negative correlation between RAD51 and BAL multinucleated macrophages was found for LGO at day 7 (Fig. 5), demonstrating that LGO in contrast to USGO may present some similarities to MWCNTs in terms of impact (i.e., inflammation and DNA damages).

*Correlation with oxidative stress* After exposure to MWCNTs, along the inflammation, a rapid response to oxidative stress occurred in the form of an upregulation of mRNA expression for HSP70 and HO1 (significant at days 1 and 7, and then going back to baseline by day 28; Additional file 1: Fig. S7; [26]), whereas SOD1 and GSH expression levels were downregulated compared to the negative control (not significant for SOD1, but significant for GSH, at days 1 and 7; Additional file 1: Fig. S7). At day 1, HSP70 expression levels were positively correlated with inflammatory markers (SAA3 and IL6; Fig. 5), as was the expression levels of SOD2 with SAA3, or the expression levels of GSH with IL1β, confirming the strong link between oxidative stress and inflammation in the impact of MWCNTs, as previously described [60]. In addition, a positive correlation between the mRNA expression of RAD51 with some oxidative stress markers (such as HO-1 at day 1, 28 and 84; or SOD2 at day 1) was found (Fig. 5). There was also a positive correlation between LIG4 and SOD1 at day 84 (Fig. 5). Taken together these results support the idea that MWCNT-induced DNA damages and their repair were related to inflammation-related oxidative stress and the regulation of ROS levels. On the other hand, there was a negative correlation between the mRNA expression level of LIG4 and HO1 at day 7 and 84 (Fig. 5), as well as between expression level of OGG1 and HO1 at day 7 (Fig. 5). Interestingly, the mRNA expression levels of the DNA repair protein OGG1 were downregulated at all tested time points (significant at day 1, 7, 28 but not 84; Additional file 1: Fig. S5). However, there was no correlation with any of the endpoints tested here (Fig. 5). Since OGG1 acts on the excision of oxidized nucleotides due to ROS in the DNA repair process, we expected to observe an increase of this marker to counteract the ROS-induced DNA damages after repeated exposure to MWCNTs (Fig. 3A). However, the only significant correlation found between this DNA repair marker and any marker associated to the oxidative stress response was at day 28 and for HSP70. Therefore, the decrease in OGG1 expression are likely explained by other feedback loop regulations not investigated here.

#### Predicting DNA damages in function of GO aspect ratio

Owing to the correlation analysis performed here, subtle variations in oxidative stress and inflammation could be associated to ongoing DNA repair mechanisms (i.e., indirect indicators of DNA damages), although some markers were not expressed significantly. We found clear correlations between DNA repair mechanisms and oxidative stress markers, as well as with secretion of pro-inflammatory mediators, or the presence of neutrophils and multinucleated macrophages in BALF. Taken together, the correlation analysis results were therefore suggesting the key role of inflammation and oxidative stress in driving the in vivo genotoxicity of GO, as reported before in vitro and discussed above. Despite the lack of comparative in vivo studies demonstrating a link between inflammation and genotoxicity, several inhalation studies have confirmed that graphene based materials (GBMs) can induce inflammation. For instance, Ma-Hock et al*.* have shown a dose-dependent increase in the number of BALF cells (especially neutrophils and lymphocytes) and LDH release, as well as an increase in the BALF concentrations of pro-inflammatory mediators at both 7 and 28 days after acute nose-only inhalation (6 h/day for 5 days) of graphene nanoplatelets (up to 10 µm; mean median aerodynamic diameter < 400 nm) in mice [61]. A series of studies using the pharyngeal aspiration or tracheal instillation routes have also reported various patterns of inflammatory response to GO [62, 63] or graphite nanoplates [64]. On the other hand, no evident lung inflammation tested for up to 21 days after exposure to GO (0.5–5 µm lateral dimension; mass median aerodynamic diameter (MMAD) of 134 nm) was found after a short-term nose-only inhalation study (6 h/day for 5 days) in rats [65]. Similarly, after chronic nose-only inhalation (6 h/day, 5 days per week, for 28 days) of graphene nanoplatelets (2 µm lateral dimension; MMAD of 123 nm) in rats, no dose dependent effects on body weight, distinct lung pathology or immune cell infiltration were identified at any time points (up to 90 days after exposure) [66]. In this study, the lack of inflammation was interestingly matched with a lack of genotoxicity, with an absence of DNA damage measured by comet assay [66], supporting the idea that inflammation and genotoxicity may work hand in hand.

In addition to a correlation between inflammation, oxidative stress and genotoxicity, our findings were also underlining an important difference between USGO and LGO. For USGO, correlations between DNA repair and oxidative stress were identified only at the earliest time point and for up to 7 days, although correlations with pro-inflammatory cytokine secretion were still measured at day 84. For LGO, we found correlations at all tested time points (up to 84 days) for both oxidative stress and inflammation events. Interestingly, MWCNT results showed absence of correlation between DNA repair and inflammatory events before day 28, but strong correlation with oxidative stress from day 1 till day 84. Taken together, these results highlight the importance of prolonged oxidative stress (and likely ROS production) on DNA damage and repair mechanisms, which may explain the presence of DNA damages at day 84 for LGO, but not for USGO. Taking into account the size difference between the two GOs, these results were also suggesting that lateral dimensions may be an essential driver of the initial response to GO materials leading to different patterns of toxicological profile.

With respect to mechanisms of toxicity, the differences between USGO and LGO in terms of repair mechanisms and pace of tissue recovery could explain the increased impact of LGO on DNA. On the one hand, USGO sheets due to their nanometric dimensions were promptly internalized by immune cells and cleared from the lungs [26]. Hence they did not elicit a strong and long-lasting inflammatory response or oxidative stress, but led only to short-term repairable genotoxic effects (Fig. 3 and Additional file 1: Fig. S8). On the other hand, LGO sheets, due to their micrometric dimensions, were entrapped in granulomatous formations inside multinucleated macrophages [26, 67] and could not be eliminated rapidly from the lungs. This was evidenced by the presence of positive Raman signals in the lung sections at 84 days [26] and the persistence of multinucleated macrophages in the BALF at the same time point (Additional file 1: Fig. S6). This could explain the long-lasting inflammation [26] and long-term bystander genotoxic effects found in the LGO high dose group (Fig. 3E, F and Additional file 1: Figs. S4A, S8). Persistence of multinucleated macrophages in lung granulomas has been reported previously for nanomaterials [68] and is often attributed to the dimensions of the considered nanomaterials. In particular, the phagocytosis capacity of macrophages is reduced in presence of high aspect ratio materials, hence requiring the fusion of these macrophages to allow appropriate internalization [26]. Therefore, it can be inferred that the long-lasting LGO-induced DNA damages could be ascribed to both the persistence of materials in the lungs and the persistent cell-mediated inflammation and oxidative stress resulting from their presence. And it appears that the geometric dimensions of LGO in comparison to USGO were the primary drivers of the material persistence, enhanced inflammation and oxidative stress, and ultimately DNA damages.

Overall, the results of the present study and our previous works [26, 27, 41] are all pointing at the importance of geometric dimensions in relation to the severity of the lung response, with GO materials of larger geometric dimensions causing the most adverse effects. Interestingly, similar conclusions were reached previously for MWCNTs [69] and single-walled CNTs [10], two high aspect ratio materials, well-known for their ability to agglomerate and their bio persistence in lung parenchyma. The present findings are also in agreement with several in vivo pulmonary studies in which GBMs of varying lateral dimensions were compared to assess the importance of GBM dimension in respect to their effect on lungs. Using either small GO (100–200 nm) or large GO (800–1100 nm) sheets, Ma et al. reported a more pronounced inflammatory response with large GO than with small GO in lungs or BAL fluids, in particular for cytokine expression levels, 72 h after intra-tracheal instillation [62]. Similarly, Roberts et al. demonstrated that a high dose of either 20 µm or 5 µm graphite nanoplates (8–25 nm thick) was more potent than < 2 µm graphite nanoplates to induce lung inflammation after pharyngeal aspiration [64]. In addition, none of these graphite nanoplates, regardless of their geometric dimension, were able to induce fibrosis at high dose, or able to trigger any adverse response at low dose [64]. A dimension-dependent response was also identified when comparing small GO (179 nm) with large GO (1676 nm) sheets in mice after oro-pharyngeal aspiration [63]. In this case, small GO induced a higher IL1 β secretion than large GO at day 2, but the difference between the two GOs and the negative control was negligible by day 6 and up to day 21 after administration. Importantly, both GO materials were inducing TGF β secretion at day 21 after exposure, with large GO inducing a more robust response than small GO, and this translated into collagen deposition for both GO types, with the lungs of large GO exposed mice showing clear evidence of pulmonary fibrosis [63]. Of note, the large GO tested in this study was inducing a greater extend of pulmonary fibrosis than quartz, used as positive control and a well-known lung carcinogen [63]. Even though none of these in vivo studies have directly investigated the possible impact of the GBM induced inflammation on DNA integrity, it is probable that the largest materials had the ability to induce genotoxicity, especially for those materials inducing chronic inflammation. In respect of the present study, future work should aim at determining whether the long-term DNA damages found after LGO exposure can be repaired at later time points (e.g., > 3 months) or lead to mutation and/or cancer. This is particularly important considering that these DNA damages were associated with an acute neutrophil response (Additional file 1: Fig. S6) that has been shown to increase the risk of carcinogenesis [38].

## Conclusions

DNA damages induced by GO sheets in mouse lung tissues after pulmonary exposure appear dependent on the material lateral dimensions, dose and exposure regimen. The GO sheets with nanometric dimensions (USGO) did not induce persistent DNA damages, irrespective of dose or chronicity. On the contrary, GO sheets with micrometric dimensions (LGO) triggered long-term DNA double strand breaks after repeated exposure to a high dose. Interestingly, a quick recovery was measured after single exposure to the same high dose of these larger GO sheets, highlighting the key role of exposure chronicity in the development of durable DNA damages. Combining multiparametric data analysis and spatial location of the DNA damages, we were able to reveal that inflammation and oxidative stress were two mechanisms involved in either recovery from or persistence of lung DNA damages. For nanometric GO, the recovery from DNA damages was strongly associated with the recovery from acute inflammation. On the other hand, the continuous presence of multinucleated macrophages that are linked to the persistence of micrometric GO in the lungs, was associated to DNA damages after 3 months. In summary, the present work provides some foundations towards a better understanding of the key elements (causes and mechanisms) guiding the development and/or resolution of DNA damages in mouse lungs after exposure to GO sheets. In particular, it emphasizes the importance of their lateral dimensions, which will be a crucial consideration for the risk classification of these materials and towards designing safer two dimensional materials for future commercial applications.

## Experimental section

### Preparation of carbon nanomaterial suspensions

Aqueous suspensions of ultrasmall (USGO) and large (LGO) GO sheet were produced in house, as previously described [42]. GO stock suspensions prepared in sterile water for injection, with neutralised pH, were stored at 4 °C until further use. Detailed information about the characterization of these materials can be found in Loret et al*.* [26]. Multi-walled carbon nanotube powder (MWCNTs, Mitsui-7, kind gift from Prof Ulla B. Vogel, National Research Centre for the Working Environment, Denmark) was heated in oven (150–180 °C) overnight to remove potential microorganisms. Aqueous suspension of MWCNTs was achieved by re-suspended the MWCNT powder in sterile water for injection containing 0.5% bovine serum albumin (Gibco, Thermo Fisher Scientific) and using bath sonication (VWR essential) for 5–7 min at nominal 80 W. MWCNT suspensions were prepared the day before administration to animals and then stored at 4 °C until further use. Nanomaterials contamination by endotoxins was evaluated according to Mukherjee et al. [70] and all the nanomaterials tested negative (data not shown).

### Single and repeated exposure of animals to nanomaterials

Female mice C57BL/6 J, 6–8 weeks old, were purchased from Envigo, UK. The experiments were carried out in the University of Manchester's Biological Services Facility where animals had access to water and food ad libitum. Mice were housed in ventilated cages (4 per cage) in a room with appropriate light cycle, temperature and humidity. Prior to the exposure, animals were anaesthetized in an inhalation chamber using a mixture of isoflurane (4%) and oxygen (96%) at 2 L min^−1^ flowing rate for 5 min. Animals were then held in supine position in order to deliver 30 µL of the material suspension by oro-pharyngeal aspiration. After anaesthesia recovery, mice were put in randomized cages. The administered dose for the single exposure study was 30 µg for each nanomaterial, then animals were housed for 1, 7 or 28 days (*n* = 3). For the repeated exposure study, the administered dose was either 1 or 10 µg of nanomaterials per animal, performed three times with a 14 day washout period between administrations. After the last exposure, animals (*n* = 6) were housed for 1, 7, 28 or 84 days. In both studies, water for injection (30 µL) was used as negative control while exposure to MWCNTs was the positive control for the endpoints considered in the present work. Procedures were performed after ethical approval from the UK Home Office, under the Project License no. P089E2E0A, and in accordance with ARRIVE 2.0 guidelines for animal research reporting [71].

### Lung tissue collection and processing

Mice were culled by overdose with intraperitoneal injection of Pentobarbitone. The right lungs were cut in pieces and then stored in microtubes containing either 1 mL of RNAlater Storage Solution (Merck Sigma) or 0.5 mL of RIPA Buffer (Merck Sigma), for RNA or protein extraction respectively. The left lungs were collected, washed in PBS (Gibco, ThermoFisherScientific), inflated with neutral buffered formalin solution (10%; *i.e*., 4% (v/v) formaldehyde solution), and then stored in tubes filled with 10% neutral buffered formalin solution. After 24 h, these lungs were transferred to 70% alcohol solution for histological processing. They were then embedded in paraffin and 5.0 µm thick sections were obtained using a microtome (Leica RM2255).

### Evaluation of cell phenotype and spatial location of DNA damages in lungs

DNA double-strand breaks were evaluated by immunostaining of formalin-fixed, paraffin-embedded (FFPE) lung sections with rabbit anti-mouse γ-H2A.X (phospho S139) recombinant antibodies (BLR053F, ab243906; Abcam). We performed either multiplex fluorescence immunostaining (IHC-IF) to correlate DNA damages with specific cell phenotype or peroxidase immunostaining (IHC-DAB) combined with hematoxylin and eosin (H&E) to locate the DNA damages in respect to lung immune response and material accumulation.

*For the IHC-IF*, Alexa Fluor 647 labeled donkey anti-rabbit IgG (H + L) highly cross-adsorbed secondary antibodies (Invitrogen, ThermoFisherScientific) were used to enhance the signal of the primary antibodies. In order to differentiate between DNA damages in lung alveolar epithelial cells and those in immune cells, we added either the Alexa Fluor 488 labeled rat anti-mouse/human CD324 (E-Cadherin) monoclonal antibody (clone DECMA-1, eBioscience; ThermoFisherScientific) [72] to identify epithelial cells or the Alexa Fluor 594 labeled rat anti-mouse CD45 monoclonal antibody (clone 30-F11, Biolegend) [73] to identify immune cells. Prior to immunostaining, FFPE lung sections were deparaffinized with Histo-clear (HistoChoice clearing agent; Merck Sigma), followed by re-hydration in series of ethanol and ultrapure water. Antigen retrieval was performed in sodium citrate buffer (10 mM sodium citrate, 0.05% tween 20; pH = 6) and microwave heating for 10 min. After cooling down at room temperature, the sections were permeated with Triton X-100 at 0.1% in PBS (Merck Sigma) for 10 min. Un-specific antigen blocking was performed using 10% normal goat serum (Invitrogen, ThermoFisherScientific), 1% BSA (Gibco; ThermoFisherScientific) in PBS for 2 h. Next, the sections were gently drained with a pipette to remove the blocking agent. The primary antibodies suspended in PBS with 1% BSA were then applied. The sections were incubated overnight at 4 °C. The next day, the sections were gently washed with 0.1% Triton X-100 in PBS and then incubated for 1 h with the secondary antibodies suspended in PBS with 1% BSA, at room temperature and protected from light. Sections were finally mounted using mounting medium (ProLong Gold Antifade Mountant with DAPI, Invitrogen, ThermoFisherScientific) and glass coverslips. Non-stained sections (blank) were similarly prepared in order to identify auto-fluorescence and determine fluorescence threshold before image acquisition in each channel. Seven images per animal from different parts of the lungs were acquired in a Zeiss AxioImager.D2 upright fluorescence microscope (Zeiss) using a 40×/0.5 EC Plan-neofluar objective and captured using a Coolsnap HQ2 camera (Photometrics), piloted by the μManager Software v1.4.23 (GitHub open source). Specific band pass filter sets for DAPI, FITC, Cy3 and Cy5 were used to prevent spectral bleed-through (crossover) between channels.

For* IHC-DAB*, following the incubation with the primary antibodies (rabbit anti-mouse γ-H2A.X (phospho S139) BLR053F, ab243906; Abcam) as described above, we applied horseradish peroxidase (HRP) labeled anti-rabbit IgG secondary antibodies (ABC detection IHC Kit, ab64261; Abcam), for 1 h at room temperature. Following instructions from the supplier, the DAB peroxidase substrate was then applied. In parallel to the immunostaining, H&E staining using an automatic stainer (XL autostainer, Leica Biosystems) was performed. Bright field images of H&E and IHC-DAB stained sections were generated with a slide scanner (Pannoramic 250 Flash, 3DHistech Ltd) and visualized with CaseViewer (software version 2.4.0.11902, 3DHISTECH Ltd) in order to reveal the spatial location of inflammation hot-spots, material accumulation, and DNA damages.

### Global and cell segmented analysis of DNA damages

Captured images were processed and analyzed using ImageJ software (National Institutes of Health, USA). For the cell segmentation analysis in the lung parenchyma, we used both DAPI (nucleus) and Cy5 (γ-H2A.X) masks to co-localize and count the number of nuclei positive for γ-H2A.X. In order to distinguish between cell types, channels for lung epithelial cells (CD324^+^, FTIC) and immune cells (CD45^+^, Cy3) were overlaid with DAPI and Cy5 channels. In the inflammatory areas, performing cell segmentation was not possible due to a highly compact number of DAPI positive nuclei. For this reason and to allow later comparison, a total fluorescence intensity for γ-H2A.X (Cy5) was taken per (field of view) FOV and for each animal (i.e., global DNA damage analysis). After analysis, global DNA damage data was plotted as total fluorescence intensity for γ-H2A.X (mean ± SD) per animal, and specific DNA damages in the lung parenchyma as the number of γ-H2A.X positive nuclei (mean ± SD) per animal. DNA damages in immune infiltrates were therefore reported as percentage of the total γ-H2A.X fluorescence intensity, whereas the distribution of DNA damages between epithelial and immune cells in the parenchyma was reported as percentage of total γ-H2A.X positive cells.

### Evaluation of whole lung DNA repair by multiplex RT-qPCR

The gene expression for key DNA repair enzymes (i.e., RAD51, OGG1 and Ligase IV) was assessed in lung homogenates at the various tested time points. The description of the procedures for these samples, including tissue lysis, DNA extraction, RNA isolation, synthesis of cDNA, and quantification of mRNA by Multiplex RT-qPCR can be found in Loret et al*.* [26]. The primers sequences are detailed in Additional file 1: Table S2. Gene expression values were normalized against GAPDH and were presented as fold change compared to the negative control.

### Multiparametric data correlation analysis

To identify in a time-dependent manner key biomarkers associated to the observed DNA damages in lungs repeatedly exposed to high dose of GO, we built a correlation matrix for multiple comparisons. For this purpose, we included the new data on DNA repair markers (RAD51, OGG1 and Ligase IV, expressed in log twofold change to negative control; *n* = 6; Additional file 1: Fig. S4), and our previously obtained data [26] on inflammatory markers (*n* = 6) including total number of macrophages, multinucleated macrophages and neutrophils measured in broncho-alveolar lavages, SAA3 expression level (obtained by multiplex RT-qPCR), a panel of secreted cytokines (TNF-α, IL-1α, IL-1β and IL-6; obtained by multiplex ELISA; Additional file 1: Fig. S5) and oxidative stress markers (OH-1, HSP70, SOD1, SOD2 and GSH; obtained by multiplex RT-qPCR; Additional file 1: Fig. S6). The correlation is presented as a categorical heat-map for each considered time-point, and includes the *Pearson* correlation values between − 1 (negative correlation; the two variables move in opposite directions) and + 1 (positive correlation; the two variables move in the same direction), with statistically significant *Pearson* correlation values highlighted with * (*p* < 0.05). GraphPad Prism v. 8.4.3 was used for this analysis. The raw *Pearson* R values can be found in Additional file 1: Table S3–S26.

### Statistical analysis

Raw data were imported in Graphpad Prism 8.0 (GraphPad Software Inc.) for producing graphs and performing statistical analyses. Depending on data normality (Shapiro–Wilk test), either one-way ANOVA followed by Dunnett’s post-hoc test was used to evaluate statistical differences compared to the negative control (*p < 0.05, **p < 0.01, ***p < 0.001), or Kruskall-Wallis followed by Dunn’s post-hoc test, was used to evaluate significant differences compared to the negative control at each time-point (*p < 0.05, **p < 0.01, ***p < 0.001).

## Supporting information available

Supplementary Information can be found online at: https://particleandfibretoxicology.biomedcentral.com/

## Supplementary Information

**Additional file 1**.** Figure S1**. Structural and morphological characterization of USGO and LGO. A) and B) Height AFM images, C) and D) scanning electron micrographs.** Figure S2**. Lung inflammation after single exposure to GOs. A) Immune cells infiltration and granulomatous formation in mice lungs at 1, 7 and 28 days after exposure to a single dose of carbon nanomaterials (USGO, LGO or MWCNT) compared to water for injection (= control). Lung infiltrates (Red arrow), granulomas formation (green arrows).** Figure S3**. DNA damages in lung cells after single exposure. (A) Quantification of γ-H2Ax positive nuclei of epithelial cells (E-Cadherin+) in lung parenchyma (outside inflammatory infiltrates and excluding CD45+ cells). (B) Quantification of immune cells in mice lungs (number of CD45+ cells per FOV, n = 7) with immunostaining using Alexa Fluor® 594 labelled rat anti-mouse CD45 monoclonal antibody. A single exposure to high dose of carbon nanomaterials (USGO, LGO or MWCNTs) compared to water for injection (= control). Statistical significance: *P<0.05, **P<0.01, ***P<0.001. (n = 3).** Figure S4**. DNA damages in lung epithelial cells after repeated exposure. Quantification of γ-H2Ax positive nuclei of epithelial cells (E-Cadherin+) in lung parenchyma (outside inflammatory infiltrates and excluding CD45+ cells) was performed after repeated exposure (x3) to high dose (A) or low dose (B) of carbon nanomaterials (USGO, LGO or MWCNTs), compared to water for injection (= control). Statistical significance: *P<0.05, **P<0.01, ***P<0.001. (n = 3).** Figure S5**. DNA repair activity in whole lung tissue after repeated exposure to a high dose. (A - C) mRNA expression level measured by RT-qPCR for three genes coding for DNA repair proteins, expressed as fold change, in whole lung tissue lysate after exposure to carbon materials (USGO, LGO or MWCNTs) or negative control (water for injection). Statistical significance: *P<0.05, **P<0.01, ***P<0.001. (n = 6).** Figure S6**. Published data on inflammation markers after repeated exposure to a high dose. (A - C) BALF cell counts measured after repeated exposure of mice to carbon materials (USGO, LGO or MWCNTs) or negative control (water for injection). (D) mRNA expression level measured by RT-qPCR for SAA3, expressed as fold change, in whole lung tissue lysate after exposure to carbon materials (USGO, LGO or MWCNTs) or negative control (water for injection). (E-H) Pro-inflammatory cytokines measured by ELISA multiplex. Statistical significance: *P<0.05, **P<0.01, ***P<0.001. (n = 6). From [ref 26; Loret et al, Advanced Science, 2022;9:2104559]** Figure S7**. Published data on oxidative stress markers after repeated exposure to a high dose. (A - E) mRNA expression levels measured by RT-qPCR for different genes involved in the regulation of oxidative stress, expressed as fold change, in whole lung tissue lysate after exposure to carbon materials (USGO, LGO or MWCNTs) or negative control (water for injection). Statistical significance: *P<0.05, **P<0.01, ***P<0.001. (n = 6). From [ref 26; Loret et al, Advanced Science, 2022;9:2104559].** Figure S8**. Evaluation of DNA damages after repeated exposure to a high dose of GO sheets or MWCNTs. Lung parenchyma, infiltrates, granulomatous areas and nanomaterial deposition (brown colour) are highlighted in the H&E staining. The corresponding DNA damages after immunostaining (IHC) with γ-H2AX (DAB+ nuclei) and counterstaining with haematoxylin are highlighted. The framed inset pictures represent the same area of IHC without incubation with the primary antibody. The scale bar is equivalent to 20 µm.** Table S1**. Summary of the physicochemical characterization of USGO and LGO sheets.** Table S2**. List of PCR primers.** Table S3**. Pearson R values (n = 6) for USGO, day 1.** Table S4**. P values (n = 6) for USGO, day 1.** Table S5**. Pearson R values (n = 6) for USGO, day 7.** Table S6**. P values (n = 6) for USGO, day 7.** Table S7**. Pearson R values (n = 6) for USGO, day 28.** Table S8**. P values (n = 6) for USGO, day 28.** Table S9**. Pearson R values (n = 6) for USGO, day 84.** Table S10**. P values (n = 6) for USGO, day 84.** Table S11**. Pearson R values (n = 6) for LGO, day 1.** Table S12**. P values (n = 6) for LGO, day 1.** Table S13**. Pearson R values (n = 6) for LGO, day 7.** Table S14**. P values (n = 6) for LGO, day 7.** Table S15**. Pearson R values (n = 6) for LGO, day 28.** Table S16**. P values (n = 6) for LGO, day 28.** Table S17**. Pearson R values (n = 6) for LGO, day 84.** Table S18**. P values (n = 6) for LGO, day 84.** Table S19**. Pearson R values (n = 6) for MWCNT, day 1.** Table S20**. P values (n = 6) for MWCNT, day 1.** Table S21**. Pearson R values (n = 6) for MWCNT, day 7.** Table S22**. P values (n = 6) for MWCNT, day 7.** Table S23**. Pearson R values (n = 6) for MWCNT, day 28.** Table S24**. P values (n = 6) for MWCNT, day 28.** Table S25**. Pearson R values (n = 6) for MWCNT, day 84.** Table S26**. P values (n = 6) for MWCNT, day 84.

## Data Availability

All data associated with the study are included in this article and the Supplementary Information. Raw data are available from the University of Manchester research repository https://figshare.manchester.ac.uk/ (project code # xxxxxx). GO materials are available from KK and NL upon reasonable request.

## Abbreviations

BALF: Broncho alveolar lavage fluid
DSB: Double strand breaks
E-Cad: E-cadherin
FOV: Field of view
GSH: Gluthatione S-transferase
HO-1: Heme oxygenase-1
HSP70: Heat shock protein 70
IL: Interleukin
LGO: Large GO
MWCNT: Multi-walled carbon nanotubes
Multi Mac: Multinucleated macrophages
SAA3: Serum amyloid A 3
SOD: Superoxide dismutase
SWCNT: Single-walled carbon nanotubes
TNF-α: Tumor necrosis factor-α
USGO: Ultra small GO

## References

[1] Donaldson K, Mills N, MacNee W, Robinson S, Newby D (2005). Role of inflammation in cardiopulmonary health effects of PM. Toxicol Appl Pharmacol.

[2] Arden Pope C, Dockery DW. Epidemiology of particle effects. Air Pollut Heal. 1999; 673-705. 10.1016/B978-012352335-8/50106-X.

[3] Knaapen AM, Borm PJA, Albrecht C, Schins RPF (2004). Inhaled particles and lung cancer. Part A Mechanisms Int J Cancer.

[4] Pope CA, Burnett RT, Thun MJ, Calle EE, Krewski D, Ito K (2002). Lung cancer, cardiopulmonary mortality, and long-term exposure to fine particulate air pollution. J Am Med Assoc.

[5] Jeggo PA, Pearl LH, Carr AM (2016). DNA repair, genome stability and cancer: a historical perspective. Nat Rev Cancer Nat Publ Group.

[6] Oberdörster G (2000). Pulmonary effects of inhaled ultrafine particles. Int Arch Occup Environ Health.

[7] Fadeel B, Bussy C, Merino S, Vázquez E, Flahaut E, Mouchet F (2018). Safety assessment of graphene-based materials: focus on human health and the environment. ACS Nano [Internet].

[8] Godwin H, Nameth C, Avery D, Bergeson LL, Bernard D, Beryt E (2015). Nanomaterial categorization for assessing risk potential to facilitate regulatory decision-making. ACS Nano.

[9] Knudsen KB, Berthing T, Jackson P, Poulsen SS, Mortensen A, Jacobsen NR (2019). Physicochemical predictors of multi-walled carbon nanotube–induced pulmonary histopathology and toxicity one year after pulmonary deposition of 11 different multi-walled carbon nanotubes in mice. Basic Clin Pharmacol Toxicol.

[10] Shvedova AA, Kisin E, Murray AR, Johnson VJ, Gorelik O, Arepalli S (2008). Inhalation vs. aspiration of single-walled carbon nanotubes in C57BL/6 mice: inflammation, fibrosis, oxidative stress, and mutagenesis. Am J Physiol Lung Cell Mol Physiol.

[11] Poland CA, Duffin R, Kinloch IA, Maynard A, Wallace WAH, Seaton A (2008). Carbon nanotubes introduced into the abdominal cavity of mice show asbestos-like pathogenicity in a pilot study. Nat Nanotechnol.

[12] Donaldson K, Poland CA, Murphy FA, MacFarlane M, Chernova T, Schinwald A (2013). Pulmonary toxicity of carbon nanotubes and asbestos - Similarities and differences. Adv Drug Deliv Rev.

[13] Ryman-Rasmussen JP, Cesta MF, Brody AR, Shipley-Phillips JK, Everitt JI, Tewksbury EW (2009). Inhaled carbon nanotubes reach the subpleural tissue in mice. Nat Nanotechnol.

[14] Shvedova AA, Kisin ER, Mercer R, Murray AR, Johnson VJ, Potapovich AI (2005). Unusual inflammatory and fibrogenic pulmonary responses to single-walled carbon nanotubes in mice. Am J Physiol Lung Cell Mol Physiol.

[15] Kasai T, Umeda Y, Ohnishi M, Mine T, Kondo H, Takeuchi T (2016). Lung carcinogenicity of inhaled multi-walled carbon nanotube in rats. Part Fibre Toxicol.

[16] Hojo M, Maeno A, Sakamoto Y, Ohnuki A, Tada Y, Yamamoto Y (2022). Two-year intermittent exposure of a multiwalled carbon nanotube by intratracheal instillation induces lung tumors and pleural mesotheliomas in F344 rats. Part Fibre Toxicol [Internet].

[17] Bussy C, Ali-Boucetta H, Kostarelos K (2013). Safety considerations for graphene: lessons learnt from carbon nanotubes. Acc Chem Res.

[18] Moon JJ, Xu C, Hong H, Lee Y, Park KS, Sun M (2020). Efficient lymph node-targeted delivery of personalized cancer vaccines with reactive oxygen species-inducing reduced graphene oxide nanosheets. ACS Nano.

[19] De Lázaro I, Vranic S, Marson D, Rodrigues AF, Buggio M, Esteban-Arranz A (2019). Graphene oxide as a 2D platform for complexation and intracellular delivery of siRNA. Nanoscale.

[20] De Sousa M, Visani De Luna LA, Fonseca LC, Giorgio S, Alves OL (2018). Folic-acid-functionalized graphene oxide nanocarrier synthetic approaches, characterization, drug delivery study, and antitumor screening. ACS Appl Nano Mater.

[21] Yang K, Zhang S, Zhang G, Sun X, Lee ST, Liu Z (2010). Graphene in mice: Ultrahigh in vivo tumor uptake and efficient photothermal therapy. Nano Lett Am Chem Soc.

[22] Di Cristo L, Grimaldi B, Catelani T, Vázquez E, Pompa PP, Sabella S (2020). Repeated exposure to aerosolized graphene oxide mediates autophagy inhibition and inflammation in a three-dimensional human airway model. Mater Today Bio.

[23] Drasler B, Kucki M, Delhaes F, Buerki-Thurnherr T, Vanhecke D, Korejwo D (2018). Single exposure to aerosolized graphene oxide and graphene nanoplatelets did not initiate an acute biological response in a 3D human lung model. Carbon.

[24] Spinazzè A, Cattaneo A, Campagnolo D, Bollati V, Bertazzi PA, Cavallo DM (2016). Engineered nanomaterials exposure in the production of graphene. Aerosol Sci Technol.

[25] Su WC, Ku BK, Kulkarni P, Cheng YS (2016). Deposition of graphene nanomaterial aerosols in human upper airways. J Occup Environ Hyg.

[26] Loret T, de Luna LAV, Fordham A, Arshad A, Barr K, Lozano N (2022). Innate but not adaptive immunity regulates lung recovery from chronic exposure to graphene oxide nanosheets. Adv Sci.

[27] Rodrigues AF, Newman L, Jasim D, Mukherjee SP, Wang J, Vacchi IA (2020). Size-dependent pulmonary impact of thin graphene oxide sheets in mice: toward safe-by-design. Adv Sci.

[28] Bengtson S, Knudsen KB, Kyjovska ZO, Berthing T, Skaug V, Levin M (2017). Differences in inflammation and acute phase response but similar genotoxicity in mice following pulmonary exposure to graphene oxide and reduced graphene oxide. PLoS One [Internet].

[29] Poulsen SS, Bengtson S, Williams A, Jacobsen NR, Troelsen JT, Halappanavar S (2021). A transcriptomic overview of lung and liver changes one day after pulmonary exposure to graphene and graphene oxide. Toxicol Appl Pharmacol.

[30] Chortarea S, Kuru OC, Netkueakul W, Pelin M, Keshavan S, Song Z, et al. Hazard assessment of abraded thermoplastic composites reinforced with reduced graphene oxide. J Hazard Mater. 2022;435:129053. Available from: https://linkinghub.elsevier.com/retrieve/pii/S0304389422008421 10.1016/j.jhazmat.2022.129053 35650742

[31] Yang S, Huang Y, Zhao Q (2022). Epigenetic alterations and inflammation as emerging use for the advancement of treatment in non-small cell lung cancer. Front Immunol [Internet].

[32] Van Rijt SH, Bein T, Meiners S (2014). Medical nanoparticles for next generation drug delivery to the lungs. Eur Respir J Eur Respir Soc.

[33] Williams RO, Carvalho TC, Peters JI (2011). Influence of particle size on regional lung deposition - what evidence is there?. Int J Pharm Elsevier.

[34] Mossman BT, Churg A (1998). Mechanisms in the pathogenesis of asbestosis and silicosis. Am J Respir Crit Care Med.

[35] Elespuru R, Pfuhler S, Aardema MJ, Chen T, Doak SH, Doherty A (2018). Genotoxicity assessment of nanomaterials: Recommendations on best practices, assays, and methods. Toxicol Sci.

[36] Azqueta A, Dusinska M (2015). The use of the comet assay for the evaluation of the genotoxicity of nanomaterials. Front Genet.

[37] Li M, Gu MM, Tian X, Xiao BB, Lu S, Zhu W (2018). Hydroxylated-graphene quantum dots induce DNA damage and disrupt microtubule structure in human esophageal epithelial cells. Toxicol Sci.

[38] Wculek SK, Bridgeman VL, Peakman F, Malanchi I (2020). Early neutrophil responses to chemical carcinogenesis shape long-term lung cancer susceptibility. iScience.

[39] Boccuni F, Ferrante R, Tombolini F, Natale C, Gordiani A, Sabella S (2020). Occupational exposure to graphene and silica nanoparticles. Part I: workplace measurements and samplings. Nanotoxicology.

[40] Lovén K, Franzén SM, Isaxon C, Messing ME, Martinsson J, Gudmundsson A (2021). Emissions and exposures of graphene nanomaterials, titanium dioxide nanofibers, and nanoparticles during down-stream industrial handling. J Expo Sci Environ Epidemiol.

[41] Vranic S, Rodrigues AF, Buggio M, Newman L, White MRH, Spiller DG (2018). Live imaging of label-free graphene oxide reveals critical factors causing oxidative-stress-mediated cellular responses. ACS Nano.

[42] Rodrigues AF, Newman L, Lozano N, Mukherjee SP, Fadeel B, Bussy C, et al. A blueprint for the synthesis and characterisation of thin graphene oxide with controlled lateral dimensions for biomedicine. 2D Mater. IOP Publishing; 2018;5:035020. Available from: http://stacks.iop.org/2053-1583/5/i=3/a=035020?key=crossref.1d398b3c0316deeca39e85128a3426dc

[43] Schinwald A, Murphy FA, Jones A, MacNee W, Donaldson K (2012). Graphene-based nanoplatelets: a new risk to the respiratory system as a consequence of their unusual aerodynamic properties. ACS Nano.

[44] Grosse Y, Loomis D, Guyton KZ, Lauby-Secretan B, El Ghissassi F, Bouvard V (2014). Carcinogenicity of fluoro-edenite, silicon carbide fibres and whiskers, and carbon nanotubes. Lancet Oncol.

[45] Toyokuni S. Genotoxicity and carcinogenicity risk of carbon nanotubes. Adv Drug Deliv Rev. 2013;65:2098–110. Available from: https://www.sciencedirect.com/science/article/pii/S0169409X1300149X?via%3Dihub 10.1016/j.addr.2013.05.011 23751780

[46] Relier C, Dubreuil M, Garcìa OL, Cordelli E, Mejia J, Eleuteri P (2017). Study of TiO_2_ P25nanoparticles genotoxicity on lung, blood, and liver cells in lung overload andnon-overload conditionsafter repeated respiratory exposure in rats. Toxicol Sci.

[47] Nourshargh S, Renshaw SA, Imhof BA (2016). Reverse migration of neutrophils: where, when, how, and why?. Trends Immunol.

[48] Bourdon JA, Saber AT, Jacobsen NR, Jensen KA, Madsen AM, Lamson JS (2012). Carbon black nanoparticle instillation induces sustained inflammation and genotoxicity in mouse lung and liver. Part Fibre Toxicol BioMed Central.

[49] Kato T, Totsuka Y, Ishino K, Matsumoto Y, Tada Y, Nakae D (2013). Genotoxicity of multi-walled carbon nanotubes in both in vitro and in vivo assay systems. Nanotoxicology.

[50] Oberdörster G (2010). Safety assessment for nanotechnology and nanomedicine: concepts of nanotoxicology. J Intern Med.

[51] Landsiedel R, Sauer UG, Ma-Hock L, Schnekenburger J, Wiemann M (2014). Pulmonary toxicity of nanomaterials: a critical comparison of published in vitro assays and in vivo inhalation or instillation studies. Nanomedicine.

[52] Bengtson S, Kling K, Madsen AM, Noergaard AW, Jacobsen NR, Clausen PA (2016). No cytotoxicity or genotoxicity of graphene and graphene oxide in murine lung epithelial FE1 cells in vitro. Environ Mol Mutagen.

[53] Mittal S, Kumar V, Dhiman N, Chauhan LKS, Pasricha R, Pandey AK. Physico-chemical properties based differential toxicity of graphene oxide/reduced graphene oxide in human lung cells mediated through oxidative stress. Sci Rep. 2016;6:39548. Available from: http://www.nature.com/articles/srep39548 10.1038/srep39548 PMC5175188 28000740

[54] Schins RPF, Knaapen AM (2007). Genotoxicity of poorly soluble particles. Inhal Toxicol.

[55] Burgum MJ, Clift MJD, Evans SJ, Hondow N, Tarat A, Jenkins GJ (2021). Few-layer graphene induces both primary and secondary genotoxicity in epithelial barrier models in vitro. J Nanobiotechnol.

[56] Chatterjee N, Yang J, Choi J. Differential genotoxic and epigenotoxic effects of graphene family nanomaterials (GFNs) in human bronchial epithelial cells. Mutat Res Toxicol Environ Mutagen. 2016;798–799:1–10. Available from: https://www.sciencedirect.com/science/article/pii/S1383571816300262?via%3Dihub 10.1016/j.mrgentox.2016.01.006 26994488

[57] Poulsen SS, Jackson P, Kling K, Knudsen KB, Skaug V, Kyjovska ZO (2016). Multi-walled carbon nanotube physicochemical properties predict pulmonary inflammation and genotoxicity. Nanotoxicology.

[58] Postel-Vinay S, Vanhecke E, Olaussen KA, Lord CJ, Ashworth A, Soria JC (2012). The potential of exploiting DNA-repair defects for optimizing lung cancer treatment. Nat Rev Clin Oncol.

[59] Visnes T, Cázares-Körner A, Hao W, Wallner O, Masuyer G, Loseva O (2018). Small-molecule inhibitor of OGG1 suppresses proinflammatory gene expression and inflammation. Science.

[60] Dong J, Ma Q (2016). Suppression of basal and carbon nanotube-induced oxidative stress, inflammation and fibrosis in mouse lungs by Nrf2. Nanotoxicology.

[61] Ma-Hock L, Strauss V, Treumann S, Küttler K, Wohlleben W, Hofmann T (2013). Comparative inhalation toxicity of multi-wall carbon nanotubes, graphene, graphite nanoplatelets and low surface carbon black. Part Fibre Toxicol.

[62] Ma J, Liu R, Wang X, Liu Q, Chen Y, Valle RP (2015). Crucial role of lateral size for graphene oxide in activating macrophages and stimulating pro-inflammatory responses in cells and animals. ACS Nano.

[63] Wang X, Duch MC, Mansukhani N, Ji Z, Liao Y-P, Wang M (2015). Use of a pro-fibrogenic mechanism-based predictive toxicological approach for tiered testing and decision analysis of carbonaceous nanomaterials. ACS Nano [Internet] Am Chem Soc.

[64] Roberts JR, Mercer RR, Stefaniak AB, Seehra MS, Geddam UK, Chaudhuri IS (2016). Evaluation of pulmonary and systemic toxicity following lung exposure to graphite nanoplates: a member of the graphene-based nanomaterial family. Part Fibre Toxicol.

[65] Kim YH, Jo MS, Kim JK, Shin JH, Baek JE, Park HS (2018). Short-term inhalation study of graphene oxide nanoplates. Nanotoxicology.

[66] Kim JK, Shin JH, Lee JS, Hwang JH, Lee JH, Baek JE (2016). 28-Day inhalation toxicity of graphene nanoplatelets in Sprague-Dawley rats. Nanotoxicology.

[67] Pagán AJ, Ramakrishnan L (2018). The formation and function of granulomas. Annu Rev Immunol.

[68] Trout KL, Holian A (2020). Macrophage fusion caused by particle instillation. Curr Res Toxicol.

[69] Muller J, Huaux F, Moreau N, Misson P, Heilier JF, Delos M (2005). Respiratory toxicity of multi-wall carbon nanotubes. Toxicol Appl Pharmacol.

[70] Mukherjee SP, Lozano N, Kucki M, Del R-C, Newman L, Vázquez E (2016). Detection of endotoxin contamination of graphene based materials using the TNF-α expression test and guidelines for endotoxin-free graphene oxide production. PLoS ONE.

[71] du Sert NP, Hurst V, Ahluwalia A, Alam S, Avey MT, Baker M (2020). The arrive guidelines 2.0: updated guidelines for reporting animal research. PLoS Biol.

[72] Lovisa S, LeBleu VS, Tampe B, Sugimoto H, Vadnagara K, Carstens JL (2015). Epithelial-to-mesenchymal transition induces cell cycle arrest and parenchymal damage in renal fibrosis. Nat Med.

[73] Cornet A, Savidge TC, Cabarrocas J, Deng WL, Colombel JF, Lassmann H (2001). Enterocolitis induced by autoimmune targeting of enteric glial cells: A possible mechanism in Crohn’s disease?. Proc Natl Acad Sci USA.

